# NETosis before and after Hyperglycemic Control in Type 2 Diabetes Mellitus Patients

**DOI:** 10.1371/journal.pone.0168647

**Published:** 2016-12-22

**Authors:** Agostina Carestia, Gustavo Frechtel, Gloria Cerrone, María A. Linari, Claudio D. Gonzalez, Patricia Casais, Mirta Schattner

**Affiliations:** 1 Laboratory of Experimental Thrombosis, Institute of Experimental Medicine-CONICET-National Academy of Medicine, Buenos Aires, Argentina; 2 Genetics and Molecular Biology, Department of Microbiology, Immunology and Biotechnology, School of Pharmacy and Biochemistry, University of Buenos Aires (UBA), Buenos Aires, Argentina; 3 Nutrition and Endocrinology Section, NORMED/UOM, Buenos Aires, Argentina; 4 Department of Pharmacology, School of Medicine, University of Buenos Aires (UBA), Buenos Aires, Argentina; Hospital for Sick Children, CANADA

## Abstract

**Introduction and Objective:**

Diabetes is characterized by chronic inflammation, endothelial dysfunction, increased risk of infections and early cardiovascular disease. By releasing neutrophil extracellular traps (NETs), neutrophils kill bacteria and exert pro-inflammatory and pro-thrombotic activities. Increased NETosis has been found in cross-sectional studies including treated type 2 diabetes mellitus (T2DM) patients. In this study, we determined whether the ability of neutrophils to form NETs differs in diabetic patients pre- and post-hyperglycemic control versus healthy donors (HD), and the relationship between NETosis with pro-thrombotic, pro-inflammatory biomarkers and thrombotic clinical events.

**Methods:**

Diabetic patients recently diagnosed and after 6 and 12 months of treatment (N = 25) and HD (N = 25) were included. NET formation was studied by microscopy and fluorometry. Nucleosomes, HNE-DNA complexes, von Willebrand factor (vWF), IL6 and TNFα plasma levels were measured by ELISA and P-selectin on the platelet surface was assessed by cytometry.

**Results:**

Basal levels of NETs in recently diagnosed T2DM patients were higher compared to HD. While TNFα stimulation of control neutrophils resulted in DNA release, patient neutrophils were not responsive. Although glycemia decreased after 6 months of metformin treatment, basal and TNFα and PMA-stimulated NETs reached normal values after 12 months. Compared to controls, nucleosomes, HNE-DNA complexes, IL-6 and TNFα levels were increased in recently diagnosed patients and decreased after 12 months of treatment. P-selectin and vWF levels were similar in both populations.

**Conclusion:**

Our data suggest that NETs could represent a biomarker for T2DM. Increased NETosis in T2DM patients does not appear to be the consequence of impaired glycemic control but rather due to pro-inflammatory cytokines and is not related to thrombotic events.

## Introduction

Neutrophils are highly specialized effector cells involved in host inflammatory responses and immune surveillance. They play an important role during the early host response to infection by a coordinated series of effector functions that include chemotaxis, phagocytosis and the generation of reactive oxygen species (respiratory burst) [1]. Moreover, it was recently discovered that, after activation, neutrophils release their DNA content together with granular proteins to form neutrophil extracellular traps (NETs) [2]. This process is a novel antimicrobial activity through which neutrophils can trap and kill microbes in the blood and tissue during infection.

Although NET formation was initially considered to be a host response against pathogen invasion, it has been observed that, if uncontrolled, NET formation switches from a beneficial host response into a major cause of tissue damage and organ failure [3–5]. Besides pathogens, NETs might also be triggered by cytokines or danger signals such as cholesterol crystals and, therefore, NETs are considered to be new mediators of sterile inflammation [6–9]. In fact, increasing evidence suggests that NET formation might be involved not only in sepsis but also in the pathogenesis of acute and chronic non-infectious inflammatory diseases including myocardial infarction, deep vein thrombosis and atherosclerosis [7, 9–11]. Type 2 Diabetes Mellitus (T2DM) is a chronic metabolic and inflammatory disorder that leads to the development of a number of complications, including early cardiovascular disease and an increased incidence of infections [12, 13]. The role of NETs in T2DM patients is far from being completely understood. It has recently been described that high glucose *in vitro* and hyperglycemia *in vivo* increase the release of NETs and circulating markers of NETosis, respectively [14, 15]. Moreover, the expression of peptidyl-arginine-deiminase, an enzyme important in chromatin decondensation and DNA release, is elevated in neutrophils from individuals with diabetes [16]. However, these studies were not only cross sectional but also included patients already under pharmacological treatment. Therefore, our principal aim was to evaluate the presence of NETs and the ability of neutrophils to form NETs in an inception cohort of T2DM patients with hyperglycemia at diagnosis and later when the normoglycemia was achieved after 6 and 12 months of treatment with metformin. In addition, we aimed to determine the relationship between NETosis with pro-thrombotic and pro-inflammatory biomarkers, and whether the presence of NETs is associated with thrombotic clinical events in these patients.

## Materials and Methods

### Subjects

This study was conducted according to the principles expressed in the Declaration of Helsinki and was approved by the Ethical Committee of the National Academy of Medicine, Buenos Aires, NORMED/UOM and Clinical Hospital.

The study was designed as an inception cohort. Inclusion criteria: adult patients were included at the time of T2DM diagnosis (T2DM was diagnosed according to the American Diabetes Association criteria [17]) and followed-up for 12 months. Patients with active systemic disorders, infections or under chronic treatment with acetyl salicylic acid were excluded. Treatment was initiated at diagnosis and, since this was not an interventional trial, patients received the standard care by their treating physicians. In this regard, they were treated with metformin at different doses. Results of routine physical examination (including body mass index; BMI), medications, and lab tests were recorded at baseline and after 6 and 12 months of follow-up. Thrombotic events such as myocardial infarction, angina, stroke and venous thromboembolism were documented.

Blood was drawn at diagnosis (before starting therapy) and after 6 and 12 months of diagnosis. Anthropometric measurements (height, weight, BMI and waist circumference), and systolic and diastolic blood pressure (SBP and DBP, respectively) were determined by a standardized protocol.Venous blood samples (20 mL) were obtained after a 12 h overnight fast.Fasting plasma glucose (FPG) was determined by the glucose-oxidase method (GLU Glucose GOD-PAP, Roche Diagnostics, Mannheim, Germany) in a Hitachi 727 auto-analyzer.

Total cholesterol (TC), triglycerides (TG) and high-density lipoprotein cholesterol (HDL-C) were determined in serum by standard enzymatic methods using commercial kits (TG Triglycerides GPO-PAP, CHOL Cholesterol CHOD-PAP and Phosphotungstate Precipitant, Roche Diagnostics, Mannheim, Germany) with a Hitachi 727 auto-analyzer. Intra-CV (coefficient of variation) for TG and TC were 1.3% and 1.1%, respectively. Inter-CVs were 2.4% and 1.5%, respectively. HbA1c was measured by HPLC with a commercial kit (Roche Diagnostics, Mannheim, Germany).

Control group: in order to determine NET formation, platelet and inflammation markers in the general non-diabetic population, a group of 25 healthy individuals were evaluated.

All subjects provided informed written consent for the collection of samples and subsequent analysis.

### Reagents

Thymus DNA and poly-L-Lysine were purchased from Sigma Aldrich (St. Louis, MO, USA). TO-PRO 3, SYBR Gold, anti-rabbit Alexa 546 antibody (Ab) were obtained from Invitrogen (Carlsbad, CA, USA). Rabbit anti-human neutrophil elastase (HNE) Ab was obtained from Calbiochem-Merck Millipore (Darmstad, Germany). The Ab against MPO-FITC was from Biolegend (San Diego, CA, USA). FITC-conjugated anti-human CD62P and FITC-irrelevant IgG1 were obtained from BD Biosciences (San José, CA, USA). Rabbit anti-human von Willebrand factor (vWF) was obtained from Dako (Denmark, CA, USA). Micrococcal nuclease (MNase) and the cell death detection ELISAPLUS kit were purchased from Roche Diagnostics (Mannheim, Germany). Human interleukin (IL)-6 high sensitivity ELISA was purchased from eBioscience (San Diego, CA, USA).

### Isolation of human neutrophils

Neutrophils were isolated from peripheral blood by Ficoll-Hypaque gradient centrifugation and dextran sedimentation, as described previously [18]. Cell suspensions contained 98% neutrophils, as determined by May Grunwald-Giemsa stained cytopreps, and the levels of monocyte contamination were always 0.2%, as evaluated by CD14 staining and flow cytometry (S1 Fig).

### NET formation assay

Neutrophils (5x10^5^/ml) were seeded in 24-well flat-bottom-plates with poly-L-Lysine coated coverslips, stimulated with TNFα (20 ng/ml) and placed in a humidified incubator at 37°C with CO_2_ (5%) for 180 minutes.

### Immunofluorescence assays

After TNFα stimulation, the cells were fixed with paraformaldehyde (PFA, 1%), permeabilized with 0.1% Triton-X and blocked with goat serum. Cells were then stained with mouse anti-human MPO-FITC Ab (1:20) and rabbit anti-HNE (1:1000) or the corresponding IgG controls. For HNE staining, Alexa Fluor 546-labeled goat anti-rabbit secondary Ab, DNA was stained with TO-PRO 3. Mounted specimens were analyzed by confocal fluorescence microscopy using a FV-1000 microscope (Olympus, Tokyo, Japan) equipped with a Plapon 60x/NA1·42 objective.

### Quantification of extracellular DNA

DNA released from neutrophils during NET formation was digested with MNase (500 mU/ml) for 15 minutes. EDTA (5 mM) was added to stop nuclease activity. Supernatants were collected, centrifuged and DNA was measured in the supernatants using SYBR Gold in a fluorometer (Biotek Instruments, Winooski, VT, USA). The calibration curve was constructed using a thymus DNA of a known concentration.

### Determination of nucleosome, IL-6, TNFα and vWF plasma levels

Plasma from healthy donors and diabetic patients was obtained by blood centrifugation. Plasmatic levels of nucleosomal DNA, human IL-6 and TNFα were measured using commercial ELISA kits (Cell death, ELISAPLUS Roche Diagnostic and eBioscience) and vWF levels were measured as previously described [19].

### HNE–DNA complex ELISA

Quantification of HNE-DNA complexes was performed as previously described [20]. Briefly, 96-well plates were coated with 5 μg/ml anti-HNE Ab (Calbiochem) overnight at 4°C. After washing three times, plasma samples were added with incubation buffer containing a peroxidase-labeled anti-DNA mAb (Cell Death ELISAPLUS, Roche Diagnostics, dilution 1:25). The plate was then incubated for 2 h, shaking at 300 rpm at room temperature (RT). After three washes, 100 μl of peroxidase substrate (ABTS) was added. Absorbance at 405 nm was measured after 20 min of incubation at RT in the dark. Values for soluble HNE-DNA complexes are expressed as optical density (OD).

### P-selectin expression

Aliquots of platelet rich plasma (PRP) obtained by the centrifugation of blood samples (200 x g for 15 min) were fixed and stained with a FITC-CD62P (anti-P-selectin) or an equivalent amount of isotype-matched control Ab. Samples were analyzed by flow cytometry on a FACSCalibur flow cytometer using CELLQUEST software (BD Biosciences, Franklin Lakes, NJ, USA), and the results are expressed as the percentage of positive cells.

### Statistical analysis

The nature of the distribution of quantitative variables was explored by the Shapiro-Wilk test. Qualitative data are expressed as percentages (%); quantitative data are expressed as mean ± standard error. Differences between qualitative data were assessed through the use of Chi-Square test. Differences between quantitative data (diabetic patients vs. healthy donors) were explored by using Student’s t-test. The associations between two quantitative variables were studied following the Pearson linear model (simple linear regression). The corresponding “R^2^” coefficients were obtained.

Several multiple linear regression models were used to explore the effect of diabetes on several dependent variables (NETs, nucleosomes and IL-6), adjusted for BMI.

The variation of the studied variables in diabetic subjects throughout the follow-up period was assessed by the use of repeated measures ANOVA models.

MedCalc (V16.2.0) and GraphPad softwares were used for the statistical analyses. Values of P<0.05 (two tailed) were considered statistically significant.

## Results

### Study subjects

Twenty-five T2DM patients were included. The clinical characteristics of patients and healthy donors at baseline are shown in Table 1.

**Table 1:** Patients and healthy donors’ clinical characteristics at baseline.

Variable	Healthy donors	Diabetic patients
**N**	25	25
**Male sex (N)**	17	18
**Female sex (N)**	8	7
**Age (years)**	49±2	51±2
**BMI (kg/m**^**2**^**)**	27±1	35±1*
**Glycaemia (mg/dl)**	83±5	196±18*
**HbA1c (%)**	4±0.2	9.4±0.3*
**TC (mg/dl)**	188±7	202±9
**TG (mg/dl)**	170±15	192±26
**HDL-C (mg/dl)**	63±2	41±1*
**Neutrophils (x10**^**6**^**/ml)**	5.2±0.3	4.9±0.3

* P<0.05 vs. healthy donors

After diagnosis and the first blood samples were drawn, all patients started therapy with metformin (doses ranging from 500–2500 mg/d). Most patients were receiving statins and enalapril at diagnosis. The majority of patients achieved a normoglycemic level at 6 and 12 months (80% of patients had HbA1c <7%). There were no thrombotic events during follow-up.

### Diabetic patients show higher levels of NET formation and circulating nucleosomes than healthy controls

**Fig 1:**
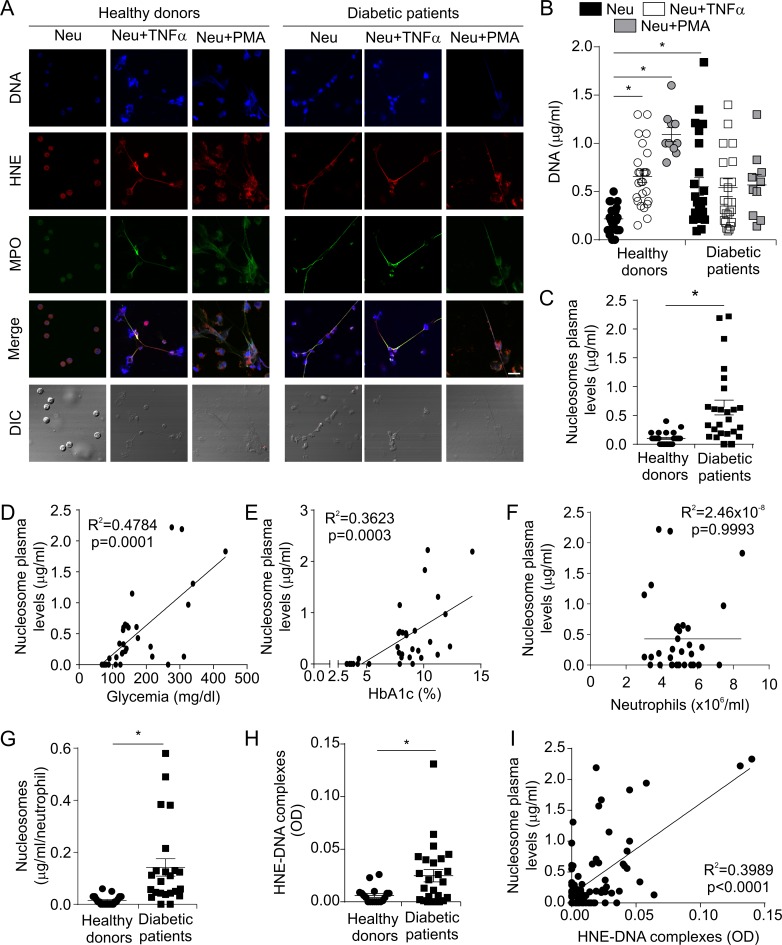
NET formation in diabetic patients. Neutrophils (Neu, 5x10^5^/ml) were isolated from healthy donors or recently diagnosed diabetic patients and stimulated or not with TNFα (20 ng/ml) or PMA (50 nM), and then the cells (A) were fixed, permeabilized and stained with TO-PRO 3 for DNA (blue), the specific markers anti-human neutrophil elastase (HNE, red) and anti-MPO (green) and visualized by confocal fluorescence microscopy (original magnification, 60X, scale bar: 20 μm, the images are representative of ten patients) or (B) treated with MNase and NET-associated DNA was quantified in the supernatants by fluorometry. (C) Nucleosome plasma levels were measured in the plasma of healthy donors or diabetic patients by ELISA. Correlation between nucleosome plasma levels and (D) glycaemia, (E) HbA1c or (F) neutrophil counts of diabetic patients. (G) Normalization of nucleosome levels to individual neutrophil counts. (H) HNE-DNA complex levels (optical density, OD) were measured in plasma of healthy donors or diabetic patients by ELISA. (I) Correlation between nucleosome levels and HNE-DNA complexes. *p<0.05. Student’s t-test was used in Panels (B), (C), (G) and (H) and the Pearson lineal model in Panels (D), (E), (F) and (I).

Considering that NET formation occurs during the inflammatory process and diabetic patients are characterized by a state of low chronic inflammation [21], we first analyzed the capability of patient neutrophils to generate these structures. Microscopic studies and DNA quantification revealed that neutrophils from recently diagnosed diabetic patients (basal samples) exhibited higher levels of NET generation than healthy donors (Fig 1A and 1B).

As NET formation can be triggered by some cytokines, including TNFα [6], and increased levels of pro-inflammatory cytokines are usually observed in diabetic patients [22], in the next experiments, we evaluated the release of NETs triggered by TNFα. As expected, the stimulation of healthy donor neutrophils with TNFα resulted in NET formation; however, patient neutrophils failed to induce DNA release upon stimulation (Fig 1A and 1B). Similar results were obtained using PMA as a NET inducer (Fig 1A and 1B).

Since neutrophils from diabetic patients had elevated levels of basal NET formation, we explored whether this phenomenon was also observed *in vivo*. To address this issue, we determined the level of nucleosomes, as an indirect measure of NETs, in the plasma of healthy donors and diabetic patients. Accordingly with the *in vitro* studies, the plasma of T2DM patients showed higher levels of nucleosomes than healthy donors (Fig 1C). The levels of nucleosomes were positively correlated with glucose levels (Fig 1D) and glycosylated HbA1c (Fig 1E), but not with neutrophil counts (Fig 1F). When the nucleosome levels were normalized to individual neutrophil counts (nucleosomes/neutrophils), a significant increase in diabetic patients was still observed compared to healthy controls, indicating that the augmented nucleosome levels were not associated with individual neutrophil counts (Fig 1G).

Since nucleosomes might be generated by means of other types of cellular death, we next analyzed the levels of plasma HNE-DNA complexes, which are specifically produced as a result of NET release [20]. Similar to nucleosome levels, T2DM patients showed increased levels of HNE-DNA complexes at diagnosis (Fig 1H). Moreover, there was a very good correlation between these complexes and nucleosome levels (Fig 1I).

### Diabetic patients have elevated levels of IL-6 and TNFα

**Fig 2:**
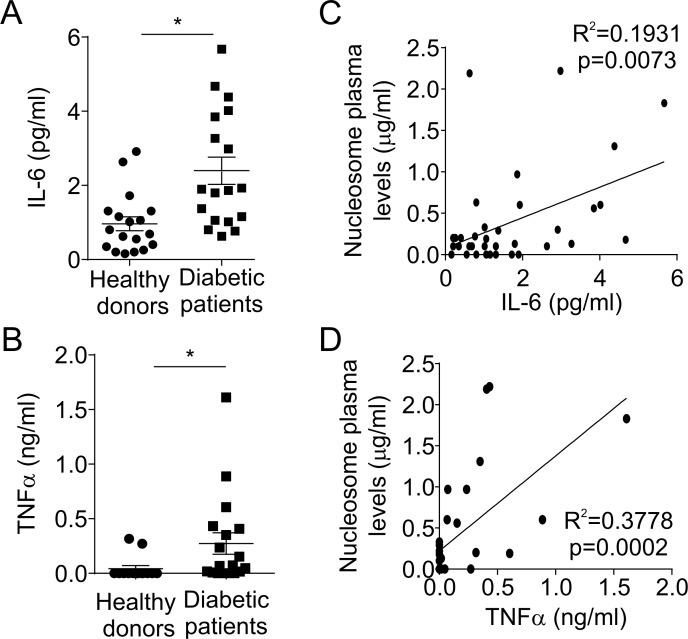
IL-6 and TNFα levels in healthy donors and diabetic patients. (A) IL-6 and (B) TNFα levels were measured by ELISA in plasma from healthy donors and diabetic patients. *P<0.05 (Student’s t-test). Correlation between (C) IL-6 or (D) TNFα and nucleosome plasma levels (Pearson linear model).

Diabetic patients usually present elevated levels of pro-inflammatory cytokines such as IL-6 and TNFα [22], which are also NET inducers [6, 14]. Accordingly, we found that diabetic patients have elevated levels of IL-6 (Fig 2A) and TNFα (Fig 2B) in plasma, which positively correlated with the levels of nucleosomes (Fig 2C and 2D).

When multiple regression models were used, diabetes remained significantly associated with NETs, nucleosomes, HNE-DNA complexes, IL-6 and TNFα after adjustment for BMI.

### Platelets and endothelium are not activated in diabetic patients

**Fig 3:**
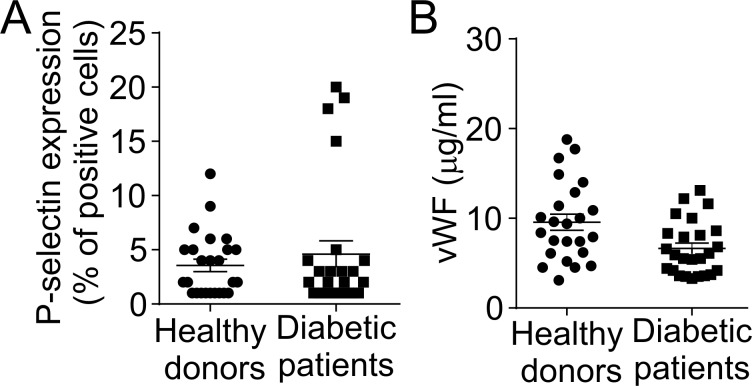
Platelets and endothelial cells are not activated in diabetic patients. (A) Platelet rich plasma (PRP) of healthy donors or diabetic patients was fixed and stained with an antibody against CD62P (P-selectin) or the corresponding IgG and P-selectin expression was measured by flow cytometry. (B) Plasma levels of vWF of healthy donors or diabetic patients were measured by ELISA (Student’s t-test).

The endothelium and platelets are critical components of intravascular NET formation [23]. To determine if there is a correlation between NET formation and platelet and endothelial activation, in the next experiments, we analyzed the expression of P-selectin and von Willebrand factor (vWF), two pro-inflammatory molecules that are exposed or released from platelets and endothelial cells, respectively, upon cell activation [11, 24]. Fig 3A and 3B show that the expression of P-selectin on the platelet membrane as well as the levels of circulating vWF were similar in diabetic patients and in healthy donors.

### NET formation and cytokine production in metformin-treated patients

**Fig 4:**
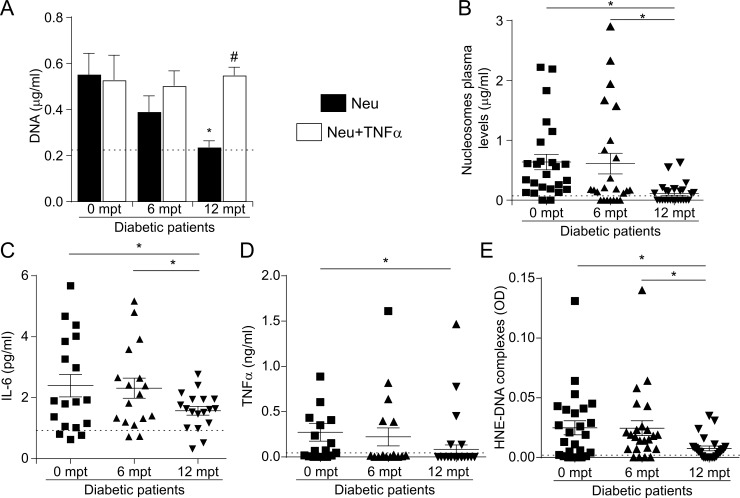
Effect of metformin treatment in diabetic patients. (A) Neutrophils (Neu, 5x10^5^/ml) were isolated from diabetic patients recently diagnosed (0 months post treatment, mpt) and 6 or 12 mpt and stimulated or not with TNFα (20 ng/ml), and then the cells were treated with MNase and NET-associated DNA was quantified in the supernatants by fluorometry. *P<0.05 vs. Neu of diabetic patients 0 mpt; #P<0.05 vs. Neu of diabetic patients 12 mpt. Plasma levels of (B) nucleosomes, (C) IL-6, (D) TNFα and (E) HNE-DNA complexes were measured in plasma of diabetic patients at 0, 6 or 12 mpt by ELISA. *P<0.05 (repeated measures ANOVA). Dotted line indicates mean value in healthy donors.

To evaluate the effect of metabolic control, we re-evaluated NETosis and cytokine production in diabetic patients after metformin treatment. Interestingly, although the glucose/HbA1c levels of diabetic patients were restored to normal values after six months of metformin treatment (Table 2), the basal formation of NETs, the failure of neutrophils to form these DNA structures after TNFα stimulation, as well as the presence of nucleosomes and HNE-DNA complexes in the plasma of diabetic patients were still observed (Fig 4A and 4B). However, all these patient neutrophil functional responses returned to normal values after 12 months of treatment (Fig 4A and 4B). Of note, neutrophil counts did not change with treatment (Table 2).

**Table 2:** Characteristics of diabetic patients at baseline (0) and 6 and 12 months post treatment (mpt).

Variable	Diabetic patients		
	0 mpt	6 mpt	12 mpt
**Glycaemia (mg/dl)**	196±18	119±6*	114±3*
**HbA1c (%)**	9.4±0.3	6.4±0.2*	6.1±0.2*
**TC (mg/dl)**	202±9	206±10	191±6
**TG (mg/dl)**	192±26	208±23	170±21
**HDL-C (mg/dl)**	41±1	42±2	45±2
**Neutrophils (x10**^**6**^**/ml)**	4.9±0.3	5.5±0.4	5.2±0.2

* P<0.05 vs. diabetic patients 0 mpt.

**Fig 5:**
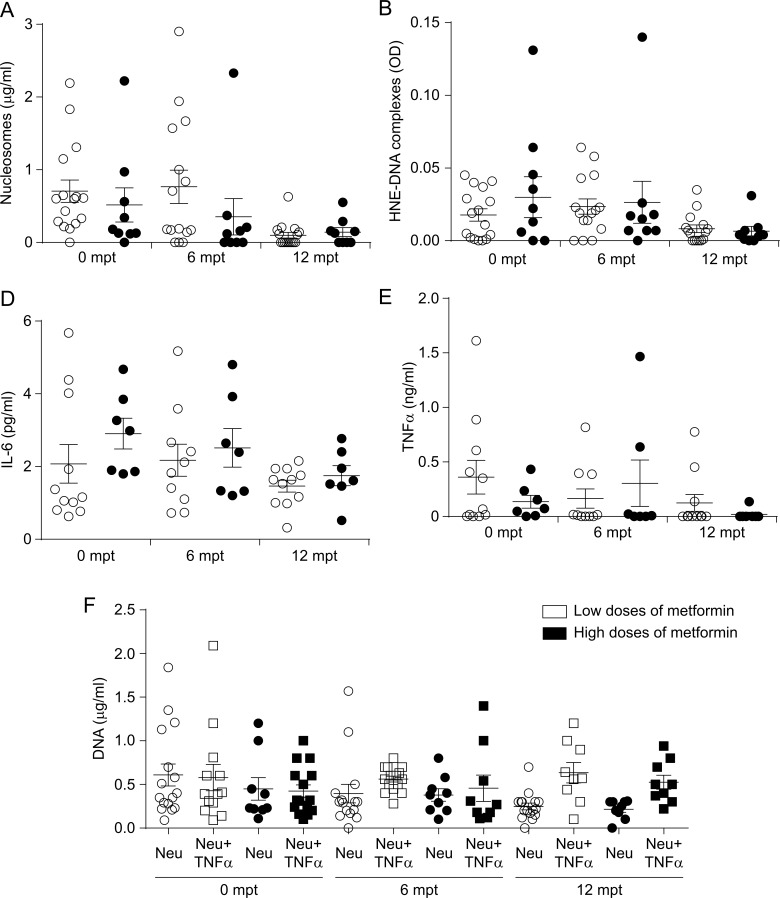
Effect of low (<1500 mg) and high (>1500 mg) metformin doses. T2DM patients were divided in two groups: patients treated with low or high doses of metformin. A) Nucleosomes, B) HNE-DNA complexes, C) IL-6, D) TNFα and E) *in vitro* NET formation were compared between groups.

Similar to the ability of neutrophils to form NETs, IL-6 and TNFα levels were still significantly higher six months after metformin treatment, but started to decrease after 12 months of pharmacological therapy (Fig 4C and 4D). Interestingly, because the patients were on different doses of metformin treatment, we re-analyzed the data according to the dose regime. Since the maximal metformin dose was 2500 mg/day, we considered a cut-off of 1500 mg/day for the low and high doses; 1500 mg/day is the standard dose required in pharmacological studies evaluating a second drug in T2DM patients already treated with metformin [25]. Although the mean value of the nucleosome levels in the high metformin dose group of T2DM patients was lower than that of the low dose group, they were not statistically different (Fig 5A). In addition, there were no differences in IL-6, TNFα, HNE-DNA complexes and *in-vitro* NET formation (DNA) between the two metformin doses (Fig 5).

## Discussion

In this study, we demonstrated that neutrophils from T2DM patients form NETs without stimulation and do not respond to further activation with TNFα. Diabetic patients had increased levels of nucleosomes, HNE-DNA complexes, IL-6 and TNFα in plasma, and all neutrophil responses were restored to normal after 12 months of metformin treatment.

A basal increase in NETosis was previously described in both diabetic patients and under hyperglycemic conditions *in vitro* [14–16, 26, 27]. Interestingly, Joshi *et al*. and Menegazzo *et al*. found that, in addition to this active state, the cells did not respond to LPS [14, 15]. Our observations of neutrophil unresponsiveness to TNFα and PMA support and further extend the notion that T2DM neutrophils exhibit increased basal NET formation and are unable to further undergo NETosis. In contrast to this hypothesis, Wong *et al*. demonstrated that neutrophils of diabetic patients were more susceptible than healthy controls to NETosis when stimulated with the calcium ionophore ionomycin [16]. However, these differences could be associated with the nature of the stimulus employed in each study. While ionomycin activates neutrophils through a receptor-independent mechanism, the response to TNFα is receptor-dependent. Thus, it could be possible that TNFα receptors in T2DM neutrophils might be unavailable, either due to a decrease in their expression or saturation due to the high circulating TNFα levels usually found in diabetic patients [22]. However, the observation that NET formation with PMA was also blunted suggests that a more general signaling pathway involved in NETosis might also be altered in diabetic patients.

Besides TNFα, another cytokine that is elevated in diabetic patients is IL-6, which can also induce NET formation [14]. We not only confirmed that diabetic patients had elevated levels of both IL-6 and TNFα in plasma, but also demonstrated that these cytokines and the increased NETosis observed at diagnosis decreased after metformin treatment, suggesting that IL-6 and TNFα could be implicated in the increased levels of constitutive NETosis observed in the neutrophils of these patients.

In line with our *ex vivo* studies, we also found that T2DM patients have significantly higher levels of circulating nucleosomes than healthy subjects. The presence of NETosis in diabetic patients has been reported by other groups [14–16, 27]. However, in previous studies, patients were already under pharmacological treatment. Our study shows for the first time that diabetic patients at diagnosis present high levels of nucleosomes as well as HNE-DNA complexes that correlate with systemic glycaemia, IL-6, TNFα and HbA1c levels. Remarkably, although nucleosomes might be generated by types of cellular deaths other than NETosis, in T2DM patients, nucleosome levels appear to be the result of NET formation, suggesting that these rapid ELISA tests could be used as another biomarker for T2DM patients.

In contrast, it has been recently reported that serum levels of elastase and proteinase 3, two proteins present in NETs, are significantly reduced in Type 1 diabetes patients and are correlated with a reduction in neutrophil counts [28]. In our study, neutrophil counts did not differ between control subjects or patients and the amount of nucleosomes/neutrophil in T2DM patients was higher than control subjects, ruling out the idea that the augmented nucleosomes levels observed in T2DM patients were associated with an increased neutrophil number.

Intravascular NET formation usually occurs on the vascular endothelium, and platelets amplify this phenomenon [23]. Since both endothelial cell and platelet activation have been reported in T2DM patients, we aimed to determine whether there was a correlation between the formation of NETs and the components of this triad. Surprisingly, neither the expression of P-selectin nor the levels of vWF (as markers of platelet and endothelial cell activation, respectively) were increased in diabetic patients compared to control subjects. Although our data indicate that platelet or endothelial cell activation are not triggers of NET formation in diabetic patients, it could be possible that other more reliable cell activation markers of this process should be assessed.

It has been demonstrated that improving glycemic control in patients ameliorates immune function. For example, the efficiency of intracellular killing of microorganisms improves with better glycemic control [29]. Moreover, it has been suggested that elevations in blood glucose are in part responsible for the increased susceptibility of diabetic neutrophils to undergo NETosis [14–16]. Our present data show that although glycemic levels were restored to normal values after six months of metformin treatment, neutrophils from diabetic patients still exhibited constitutive NETosis, did not respond to TNFα and had higher levels of circulating nucleosomes and HNE-DNA complexes than normal subjects. Only after 12 months of treatment did all these parameters return to control values. The observation that, despite the normalization of glucose levels, NETosis (*in vitro* and *in vivo)* remained augmented point out that these events are not the consequence of impaired glycemic control. Of note, similar kinetics were observed for IL-6 and TNFα production, indicating that inflammation rather than the metabolic state regulates NETosis. The discrepancy of nucleosomes, HNE-DNA complexes, IL-6 and TNFα versus glucose and HbA1c levels at 6 months after treatment was unexpected, and we can only speculate that: a) the kinetics of the molecules involved might be responsible even though, as far as we know, there is no evidence in the literature; b) a cellular response might take longer than the effect of a pharmacological agent to normalize a metabolic parameter; c) other molecules not evaluated in our study or yet unknown might play a role in this complex response.

Interestingly, and in support of our hypothesis that NETosis is not a consequence of impaired glycemic control in T2DM, it has been recently reported that metformin can inhibit PMA-induced NET formation [30]. Whether these observations are due to a direct effect of metformin requires further investigation. In a recent study of well controlled-T2DM patients, Miyoshi *et al*. observed that DNA-MPO complexes in patients treated with more than three antidiabetic drugs were higher than in patients treated with fewer than three drugs [27]. In contrast, we did not observe differences between nucleosome levels or HNE-DNA complexes regarding the metformin dose. These differences could be explained by the different patient population (recently diagnosed T2DM patients and only one antidiabetic drug in our study). NETosis has been identified in the last decade as a novel form of programmed cell death occurring in neutrophils and induced by infectious agents and other inflammatory triggers [6, 23, 31]. NETs provide a natural defense by entrapping microbes [2, 23]. However, excessive or dysregulated NETosis also promotes thrombosis, inflammation and endothelial dysfunction [3, 4, 32], which may contribute to diabetic complications. The clinical implications of these observations remain to be determined. In our study, we observed neither increased platelet (P-selectin) or endothelial (vWF) activation markers nor thrombotic events during the first year of therapy. Since all the patients achieved metabolic control and decreased NETosis during therapy, the prognostic value of NETs could not be evaluated.

In conclusion, our data suggest that NETs could represent a novel biomarker for T2DM and that increased *in vivo* NET formation appears not to be the consequence of impaired glycemic control and is not associated with thrombotic events.

## Supporting Information

S1 Fig Purity of neutrophil isolation. After neutrophil isolation, cells were stained for 20 min with CD14-PE.
(TIF)

## References

[1] Amulic B, Cazalet C, Hayes GL, Metzler KD, Zychlinsky A. Neutrophil function: from mechanisms to disease. Annual review of immunology. 2012;30:459–89. 10.1146/annurev-immunol-020711-074942 22224774

[2] Brinkmann V, Reichard U, Goosmann C, Fauler B, Uhlemann Y, Weiss DS, et al Neutrophil extracellular traps kill bacteria. Science. 2004;303:1532–5. 10.1126/science.1092385 15001782

[3] Czaikoski PG, Mota JM, Nascimento DC, Sonego F, Castanheira FV, Melo PH, et al Neutrophil Extracellular Traps Induce Organ Damage during Experimental and Clinical Sepsis. PloS one. 2016;11:e0148142 10.1371/journal.pone.0148142 26849138 PMC4743982

[4] Carmona-Rivera C, Zhao W, Yalavarthi S, Kaplan MJ. Neutrophil extracellular traps induce endothelial dysfunction in systemic lupus erythematosus through the activation of matrix metalloproteinase-2. Annals of the rheumatic diseases. 2015;74:1417–24. 10.1136/annrheumdis-2013-204837 24570026 PMC4143484

[5] Kaplan MJ, Radic M. Neutrophil extracellular traps: double-edged swords of innate immunity. Journal of immunology. 2012;189:2689–95. 10.4049/jimmunol.1201719 PMC3439169 22956760

[6] Lapponi MJ, Carestia A, Landoni VI, Rivadeneyra L, Etulain J, Negrotto S, et al Regulation of neutrophil extracellular trap formation by anti-inflammatory drugs. The Journal of pharmacology and experimental therapeutics. 2013;345:430–7. 10.1124/jpet.112.202879 23536315

[7] Maugeri N, Campana L, Gavina M, Covino C, De Metrio M, Panciroli C, et al Activated platelets present high mobility group box 1 to neutrophils, inducing autophagy and promoting the extrusion of neutrophil extracellular traps. Journal of thrombosis and haemostasis: JTH. 2014;12:2074–88. 10.1111/jth.12710 25163512

[8] Carestia A, Kaufman T, Rivadeneyra L, Landoni VI, Pozner RG, Negrotto S, et al Mediators and molecular pathways involved in the regulation of neutrophil extracellular trap formation mediated by activated platelets. Journal of leukocyte biology. 2016;99:153–62. 10.1189/jlb.3A0415-161R 26320263

[9] Warnatsch A, Ioannou M, Wang Q, Papayannopoulos V. Inflammation. Neutrophil extracellular traps license macrophages for cytokine production in atherosclerosis. Science. 2015;349:316–20. 10.1126/science.aaa8064 26185250 PMC4854322

[10] von Bruhl ML, Stark K, Steinhart A, Chandraratne S, Konrad I, Lorenz M, et al Monocytes, neutrophils, and platelets cooperate to initiate and propagate venous thrombosis in mice in vivo. The Journal of experimental medicine. 2012;209:819–35. 10.1084/jem.20112322 22451716 PMC3328366

[11] Duerschmied D, Bode C, Ahrens I. Immune functions of platelets. Thrombosis and haemostasis. 2014;112:678–91. 10.1160/TH14-02-0146 25209670

[12] Fernandez-Real JM, Pickup JC. Innate immunity, insulin resistance and type 2 diabetes. Diabetologia. 2012;55:273–8. 10.1007/s00125-011-2387-y 22124608

[13] Peleg AY, Weerarathna T, McCarthy JS, Davis TM. Common infections in diabetes: pathogenesis, management and relationship to glycaemic control. Diabetes/metabolism research and reviews. 2007;23:3–13. 10.1002/dmrr.682 16960917

[14] Joshi MB, Lad A, Bharath Prasad AS, Balakrishnan A, Ramachandra L, Satyamoorthy K. High glucose modulates IL-6 mediated immune homeostasis through impeding neutrophil extracellular trap formation. FEBS letters. 2013;587:2241–6. 10.1016/j.febslet.2013.05.053 23735697

[15] Menegazzo L, Ciciliot S, Poncina N, Mazzucato M, Persano M, Bonora B, et al NETosis is induced by high glucose and associated with type 2 diabetes. Acta diabetologica. 2015;52:497–503. 10.1007/s00592-014-0676-x 25387570

[16] Wong SL, Demers M, Martinod K, Gallant M, Wang Y, Goldfine AB, et al Diabetes primes neutrophils to undergo NETosis, which impairs wound healing. Nature medicine. 2015;21:815–9. 10.1038/nm.3887 26076037 PMC4631120

[17] American Diabetes A. Standards of medical care in diabetes—2014. Diabetes care. 2014;37 Suppl 1:S14–80. 24357209 10.2337/dc14-S014

[18] Negrotto S, Malaver E, Alvarez ME, Pacienza N, D'Atri LP, Pozner RG, et al Aspirin and salicylate suppress polymorphonuclear apoptosis delay mediated by proinflammatory stimuli. The Journal of pharmacology and experimental therapeutics. 2006;319:972–9. 10.1124/jpet.106.109389 16936242

[19] Carestia A, Rivadeneyra L, Romaniuk MA, Fondevila C, Negrotto S, Schattner M. Functional responses and molecular mechanisms involved in histone-mediated platelet activation. Thrombosis and haemostasis. 2013;110:1035–45. 10.1160/TH13-02-0174 23965842

[20] Caudrillier A, Kessenbrock K, Gilliss BM, Nguyen JX, Marques MB, Monestier M, et al Platelets induce neutrophil extracellular traps in transfusion-related acute lung injury. The Journal of clinical investigation. 2012;122:2661–71. 10.1172/JCI61303 22684106 PMC3386815

[21] Wellen KE, Hotamisligil GS. Inflammation, stress, and diabetes. The Journal of clinical investigation. 2005;115:1111–9. 10.1172/JCI25102 15864338 PMC1087185

[22] Pickup JC, Chusney GD, Thomas SM, Burt D. Plasma interleukin-6, tumour necrosis factor alpha and blood cytokine production in type 2 diabetes. Life sciences. 2000;67:291–300. 10983873 10.1016/s0024-3205(00)00622-6

[23] Clark SR, Ma AC, Tavener SA, McDonald B, Goodarzi Z, Kelly MM, et al Platelet TLR4 activates neutrophil extracellular traps to ensnare bacteria in septic blood. Nature medicine. 2007;13:463–9. 10.1038/nm1565 17384648

[24] Baruch D. [Platelet—vessel wall interactions]. Therapie. 2006;61:371–8. 17243265 10.2515/therapie:2006068

[25] Wang G, Liu J, Yang N, Gao X, Fan H, Xu Y, et al MARCH2: comparative assessment of therapeutic effects of acarbose and metformin in newly diagnosed type 2 diabetes patients. PloS one. 2014;9:e105698 10.1371/journal.pone.0105698 25148570 PMC4141807

[26] Fadini GP, Menegazzo L, Rigato M, Scattolini V, Poncina N, Bruttocao A, et al NETosis Delays Diabetic Wound Healing in Mice and Humans. Diabetes. 2016;65:1061–71. 10.2337/db15-0863 26740598

[27] Miyoshi A, Yamada M, Shida H, Nakazawa D, Kusunoki Y, Nakamura A, et al Circulating Neutrophil Extracellular Trap Levels in Well-Controlled Type 2 Diabetes and Pathway Involved in Their Formation Induced by High-Dose Glucose. Pathobiology: journal of immunopathology, molecular and cellular biology. 2016;83:243–51. 10.1159/000444881 27189166

[28] Qin J, Fu S, Speake C, Greenbaum CJ, Odegard JM. NETosis-associated serum biomarkers are reduced in type 1 diabetes in association with neutrophil count. Clinical and experimental immunology. 2016;184:318–22. 10.1111/cei.12783 26939803 PMC4872375

[29] Gallacher SJ, Thomson G, Fraser WD, Fisher BM, Gemmell CG, MacCuish AC. Neutrophil bactericidal function in diabetes mellitus: evidence for association with blood glucose control. Diabetic medicine: a journal of the British Diabetic Association. 1995;12:916–20. 8846684 10.1111/j.1464-5491.1995.tb00396.x

[30] Wang H, Li T, Chen S, Gu Y, Ye S. Neutrophil Extracellular Trap Mitochondrial DNA and Its Autoantibody in Systemic Lupus Erythematosus and a Proof-of-Concept Trial of Metformin. Arthritis & rheumatology. 2015;67:3190–200. 26245802 10.1002/art.39296

[31] Fuchs TA, Abed U, Goosmann C, Hurwitz R, Schulze I, Wahn V, et al Novel cell death program leads to neutrophil extracellular traps. The Journal of cell biology. 2007;176:231–41. 10.1083/jcb.200606027 17210947 PMC2063942

[32] Fuchs TA, Brill A, Duerschmied D, Schatzberg D, Monestier M, Myers DD Jr., et al Extracellular DNA traps promote thrombosis. Proceedings of the National Academy of Sciences of the United States of America. 2010;107:15880–5. 10.1073/pnas.1005743107 20798043 PMC2936604

