# Supplementary figures and images for: NETosis before and after Hyperglycemic Control in Type 2 Diabetes Mellitus Patients
Source: PLoS One. 2016 Dec 22;11(12):e0168647. doi: 10.1371/journal.pone.0168647 (PMC5179097; doi:10.1371/journal.pone.0168647)

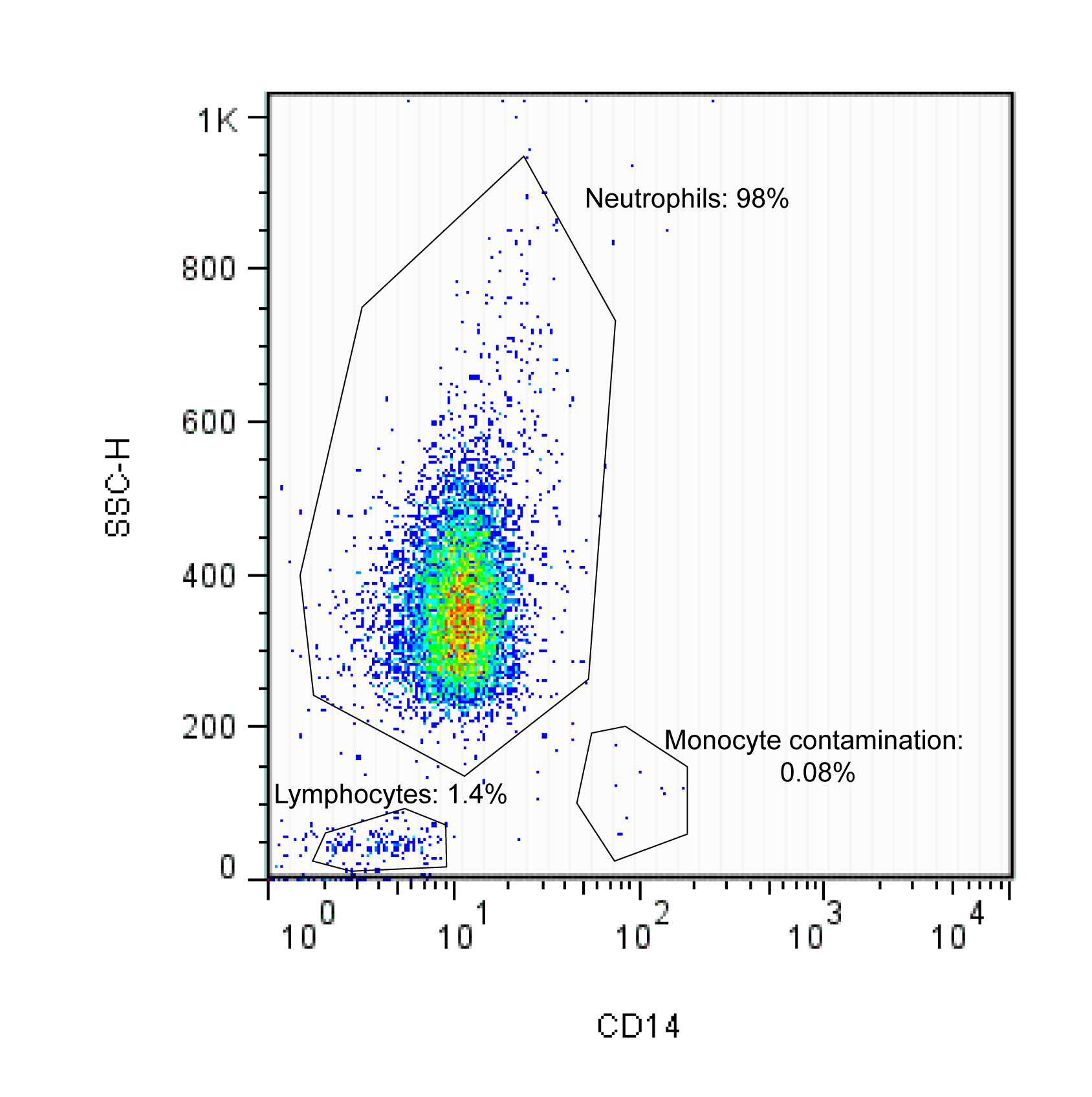

Supplement: S1 Fig — After neutrophil isolation, cells were stained for 20 min with CD14-PE. (TIF) [file pone.0168647.s001.tif]
